# Abnormal Dihydropyrimidine Dehydrogenase Activity as an Indicator of Potential 5-Fluorouracil Linked Cardiotoxicity in Colorectal Cancer Patients: Are Toxic Events Inevitable?

**DOI:** 10.7759/cureus.17712

**Published:** 2021-09-04

**Authors:** Hyginus Chakwop Ngassa, Khaled A Elmenawi, Vishwanath Anil, Harpreet Gosal, Harsimran Kaur, Lubna Mohammed

**Affiliations:** 1 Surgery, Università degli Studi di Brescia Facoltà di Medicina e Chirurgia, Brescia, ITA; 2 Surgery, California Institute of Behavioral Neurosciences & Psychology, Fairfield, USA; 3 Surgery, Cairo University, Cairo, EGY; 4 Internal Medicine, California Institute of Behavioral Neurosciences & Psychology, Fairfield, USA; 5 Internal Medicine/Emergency Medicine, Government Medical College Amritsar, Amritsar, IND; 6 Medicine, California Institute of Behavioral Neurosciences & Psychology, Fairfield, USA

**Keywords:** cardiotoxicity, colorectal cancer, 5-fu, fluoropyrimidine, capecitabine, 5-fu toxicity

## Abstract

Colorectal cancer (CRC) treatment can be limited to surgical resection for low stages of the disease while subsequent chemotherapy is the preferred treatment for the higher-stage disease. This chemotherapy relies heavily on fluoropyrimidine: 5-fluorouracil (5-FU) and capecitabine, a role played for decades. Fluoropyrimidine-linked treatment can present important and even lethal toxic events at the cardiac level like acute coronary syndrome, arrhythmias, and death.

The production of these toxic events depends on the capacity of a subject to metabolize the fluoropyrimidines adequately, and this depends on the activity of the enzyme dihydropyrimidine dehydrogenase (DPD). Any change that affects the quantity or quality of this enzyme will compromise its capacity to metabolize the fluoropyrimidines. The resultant abnormal enzyme activity exposes the patient to continuously high levels of the chemotherapeutic agent or its catabolites. Consequently, the patient becomes more susceptible to pyrimidine-linked toxic adverse events.

Genetic testing of patients for potential decreased DPD activity before subjecting them to fluoropyrimidine-based chemotherapy will help identify subjects at greater risk of increased cardiotoxicities, the possibility of prompt intervention, should these appear, and a multidisciplinary strategy aimed at managing these cases. Potential cases of cardiotoxicity in CRC patients, candidates to fluoropyrimidine toxicities, can be anticipated by pretreatment screening of DPD activity. Pretreatment screening will reduce many hospitalizations with a consequent decrease in costs both to the patients and the healthcare system.

This review article will examine the 5-FU linked cardiotoxicity, known correlated risk factors, clinical manifestations, management strategy, and the role of genetic testing in identifying high-risk patients.

## Introduction and background

Patients with advanced colorectal cancer (CRC) need to undergo adjuvant or neoadjuvant chemotherapy with fluoropyrimidines like 5-fluorouracil (5-FU), a cytotoxic antineoplastic, and antimetabolite chemotherapeutic agents. Its oral equivalent, the prodrug capecitabine, serves the same purpose. Throughout the world, both drugs represent the principal chemotherapeutic agents used to treat many solid malignancies, gastrointestinal as well as breast cancer [1,2]. Notwithstanding current advances made in management with other agents, 5-FU still constitutes the best and safest drug, whether used in isolation or with other chemotherapeutic agents [3].

Multiple studies register fluoropyrimidine-linked cardiotoxicity with varying frequencies, from 1.5% to even 30% of patients treated with 5-FU [2,4-6]. Clinical manifestations vary from asymptomatic EKG alterations, hypotension, angina, myocardial infarction, left ventricular dysfunction, heart failure, cardiogenic shock, pericarditis, dyspnea, and different arrhythmias like atrial fibrillation or ventricular tachycardia, and even ventricular fibrillation [5,6]. Saif MW et al. observed these adverse effects in subjects with cardiac comorbidities and those with no prior cardiac conditions [5]. The exact mechanism through which these drugs generate their harmful actions on the heart is not yet fully known and understood. However, some studies propose a few hypotheses that include coronary vasospasm with no occlusion of the blood vessel, which presents an elevation of cardiac enzymes [3,6]. Ehrlich et al. propose other possible mechanisms like direct damage on endothelial cells, autoimmune reactions, augmented thrombus formation in coronary vessels, and alterations in drug metabolism [3].

Dihydropyrimidine dehydrogenase (DPD) catalyzes the breakdown of over 80% metabolism of 5-FU. An inadequate activity of DPD plays a significant role in augmented cardiotoxicity [7]. Variations of the DPD gene (*DPYD*) sequence caused by numerous single-nucleotide polymorphisms (SNPs) may lead to a partial or complete deficiency of DPD activity with significant severe fluoropyrimidine toxicity [7,8]. Approximately 30-50% of patients present significant toxicities but do not have the polymorphisms. Other factors like comorbidities and multiple concurrent therapies can also play critical parts.

*DPYD* is a very polymorphic gene, and some variants such as c.1236G>A/ HapB3, c.1679T>G, c.1905+1G>A, and c.2846A>T, when deficient, produce insufficient enzyme activity with increased incidence of severe adverse drug reactions [7]. A pretreatment investigation into *DPYD* variants makes it possible to reduce the risks of adverse drug reactions. This investigation notwithstanding, significant and even lethal fluoropyrimidine linked reactions could happen at any moment during the treatments, even in patients that present no *DYPD* defects connected to the numerous SNPs [8].

This review presents a glimpse of a therapeutic approach to managing colorectal cancer with 5-FU as the critical therapeutic agent. It examines the factors responsible for potentially lethal cardiotoxicity. It emphasizes a multidisciplinary approach as a way forward to identify high-risk patients before exposure to fluoropyrimidine therapy to reduce adverse 5-FU related adverse reactions. An open question remains the eventuality of an effective screening method on all patient candidates to chemotherapeutic treatment with fluoropyrimidines like 5-FU and capecitabine. How beneficial will an efficient screening process of CRC patients be before subjecting them to chemotherapy with 5-FU compared to post-treatment hospitalization and management of patients due to complications arising from their chemotherapy regimens?

## Review

Our review article examines the published studies on CRC with its diffusion and impact worldwide. We consider fluoropyrimidine, the principal drug used in treating metastatic CRC, and its cardiotoxicity. We analyze the metabolism of said chemotherapeutic, the main enzyme involved, and its role in the previously mentioned toxicity. Are alterations in fluoropyrimidine predictable? How are cases of cardiotoxicity managed? Can pretreatment screening reduce the adverse drug reactions observed and even reduce hospitalization? Our article confronts these concerns in the following sections.

Colorectal cancer

**Figure 1:**
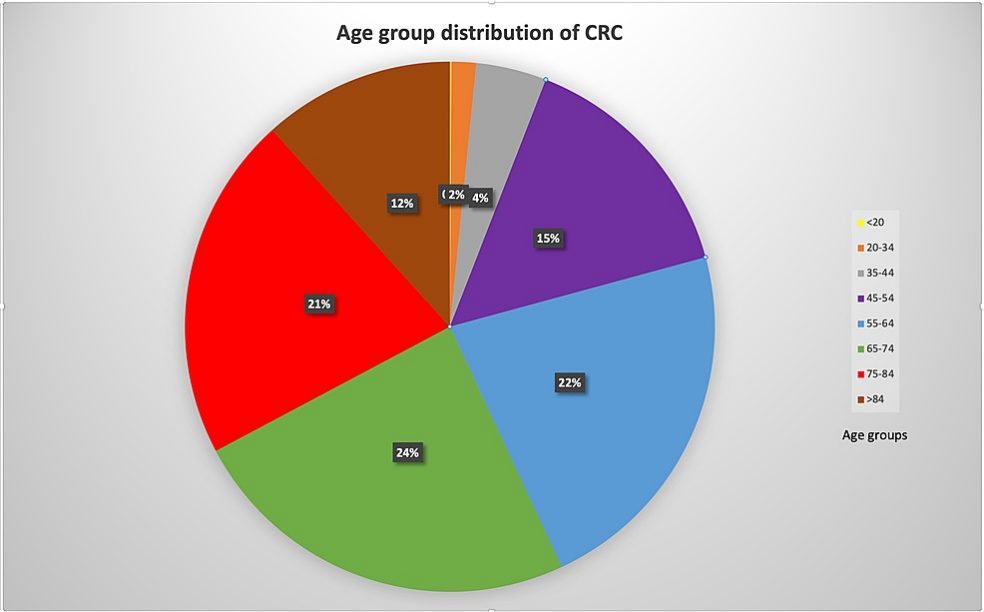
A distribution (in percentage) of new cases of colorectal cancer per age group from 2014 to 2018. Age groups in years (<20,20-34,35-44,45-54,55-64,65-74,75-84, and >85) represented by different colors. CRC: colorectal cancer Adapted from The National Cancer Institute’s Surveillance, Epidemiology, and End Results (SEER) registry [12].

CRC is among the principal reasons people die of cancer worldwide, the second most common cause for cancer-linked death in North America and Europe [1,9]. Worldwide, it's the third-most diagnosed cancer and the fourth cancer-related cause of death. The worldwide tally of CRC will probably increase by 60%, with new cases estimated at 2.2 million and possibly 1.1 million deaths by 2030 [10]. United Nations Population Fund (UNFPA) registered an increase in worldwide life expectancy in the last two decades from 64.8 years to 70 years and projected a significant increase in the elderly population globally [11]. Since many cancers are observed more in elderly subjects above 65 years of age, Itatani Y et al. hypothesize increased CRC incidence among senior subjects [10,11]. The National Cancer Institute's Surveillance, Epidemiology, and End Results (SEER) registry recorded data of subjects diagnosed with new colon cancer or rectal cancer cases from 2014 through 2018, presented in Figure 1.

About 78% of cases of CRC occur in subjects aged 55 years or above. Based on the current trend, the SEER predicts an increase in the incidence of colon cancer of 90% in subjects aged 20-34 years by 2030 and an increase of rectal cancer of 124.2%. For subjects aged 35-49 years, the SEER predicts an increase in the incidence of 27.7% for colon cancer and an increase of 46% for rectal cancer [12]. As such, combining the predictions of UNFPA, Itatani, et al. and SEER, we can expect an increase in the global population, an increase in the subpopulation of the elderly with a relative rise in cases of CRC, and also an increase in the incidence of CRC in subjects under the age of 50.

Arnold M et al. observed approximately 66% or more CRC cases in nations with elevated human development index and about 60% cases of deaths [13]. These nations invest significant resources into preventive measures, screenings, removal of eventual precancerous lesions like polyps. Their steady improvement in the management with the implementation of established guidelines on chemotherapy and radiotherapy, in various degrees, contribute to reducing a trend in death related to CRC, improving survival. and reducing cases of recurrence [13,14]. While elective surgery plays an essential role in treating low-stage CRC, both adjuvant and neoadjuvant chemoradiotherapies also play a critical in reducing CRC-related mortality, especially for higher stages of the disease [14]. This therapy relies strongly on fluoropyrimidines like 5-FU and capecitabine with regiments like leucovorin, 5-FU, oxaliplatin (FOLFOX) and oxaliplatin.

Metastatic colorectal cancer treatment

The treatment of CRC depends on the stage of the disease. Low-stage cancers need surgery alone. High-stage cancers (stage II & stage III) need adjuvant and neoadjuvant radiochemotherapy [14,15]. Table 1 shows the classifications and staging of CRC most commonly used in clinical practice, with the usual therapy applied to each stage [16].

**Table 1:** Classification of CRC and types of treatment of the various stages of cancer. *surgery is an option only in the presence of luminal occlusion of the colon/rectum. FOLFOX: leucovorin, 5-FU, oxaliplatin; CapeOx: capecitabine, oxaliplatin; T: tumor dimension (increasing size from 1-3, x being a tumor of any size); N: lymph node interested (0 meaning no lymph node involved and 1 being lymph node involved); M: the presence of distant metastasis (most frequently to the liver, lungs, and brain) Adapted from: the website of the Colorectal Cancer Association [16].

Tumor location	TNM classification	Dukes stage	Numerical stage	The prognosis (five years)	Surgical treatment	Chemotherapy, other	Radiotherapy
Limited to submucosa	T1N0M0	A	I	Above 90%	Applied	Unnecessary	Unnecessary
Muscularis mucosae	T2N0M0	B1	I	85%	Applied	Unnecessary	Unnecessary
Tunica serosa	T3N0M0	B2	II	70-80%	Applied	FOLFOX or CapeOx	Rectal cancer with 5-FU
Local lymph nodes	TxN1M0	C	III	35-65%	Applied	FOLFOX or CapeOx	Rectal cancer in association with 5-FU
Metastasis	TxN1M1	D	IV	5%	Variable*	FOLFOX, FOLFIRI, FOLFIRINOX, Irinotecan, erbitux	Rectal cancer

There is a significant improvement in the outcome of cases of metastatic CRC (mCRC) observed in the past two decades, especially in the West, with an increase in survival rates as well as a decrease in cases of local recurrence. This progress results from the fact that more and more patients benefit from preventive measures, screening, and early diagnosis and receive excellent surgical treatment, good chemotherapy regimens, or other treatments that ensure ablation [9,14,15]. More and more cases undergo a multidisciplinary approach in dedicated oncological structures explaining this progressive improvement in the outcome of mCRC; the perfecting of radiographic studies and the employment of biological markers have also offered significant contributions [9].

Fluoropyrimidines cardiotoxicity

5-FU as well as capecitabine, its prodrug, are among the fluoropyrimidines, which are the antitumoral medications employed in the treatment of solid malignant neoplasia of the gastrointestinal system affecting practically any organ from the esophagus to the anus, neoplasia of the bladder, the head, neck, and the breast [2,17,18]. The second most frequent cancer-linked death in men and women in the United States remains CRC [1,9]. Patients with high stages of the disease (stage III or higher) receive a treatment based on fluoropyrimidine as the prevailing therapy. Fluoropyrimidines still present significant adverse effects linked to its use in as many as 10-30% of patients, with a non-insignificant rate of death, varying from 0.5-1%, notwithstanding the enormous progress made in management [4,17,19]. Bone marrow suppression, diarrhea, and mucositis are some of the toxic reactions observed in 5-FU and capecitabine treatments. While the adverse effects at the heart level are less common, they are a lot more dangerous and even lethal; among these have been observed acute myocardial infarction, arrhythmias, heart failure, shock, sudden death, pericarditis, and Takotsubo cardiomyopathy [3,5,6]. Some patients present these adverse cardiac events even without a history of coronary illness. There is no clear evidence of a correlation between fluoropyrimidine-linked cardiotoxicity and the presence of cardiovascular risk factors [11,19]. The cardiac toxic effect related to fluoropyrimidines most often encountered is angina. Still, palpitations, shortness of breath, blood pressure discrepancies (increased and reduced blood pressure), general discomfort are also possible; meanwhile, less common are myocardial infarction, myocarditis, pericarditis, heart failure, and temporary cardiomyopathy [20]. Polk A et al. registered important toxic cardiac events like tachyarrhythmias (ventricular and supraventricular), coronary dissection, cardiogenic shock, and sudden cardiac death. This vast array of cardiac manifestations mentioned in literature depends on different elements like the schedule of the therapy itself, ongoing therapies, and comorbidities [20].

A precise mechanism of how fluoropyrimidine provokes toxic effects at the cardiac level is not fully understood. What seems likely is that some products of the breakdown of 5-FU and capecitabine play essential roles [20,21]. Vasospasm of the coronary vessels provoked by either 5-FU or by one of its catabolites is another possibility [3,6,21]. Still, some cardiac adverse events linked to the use of 5-FU do not seem to have a satisfactory explanation for coronary artery spasms. Patients who had previously manifested 5-FU related cardiotoxicity did not present vasospasm on angiography, even with active symptoms after reusing the drug. Besides, numerous medications that cause blood vessel dilation did not improve these adverse cardiac effects [21]. Another possible hypothesis postulated is temporary endothelial damage impeding blood vessel dilation and favoring blood coagulation from the lesions of the myocardium by catabolites of 5-FU [22].

**Figure 2:**
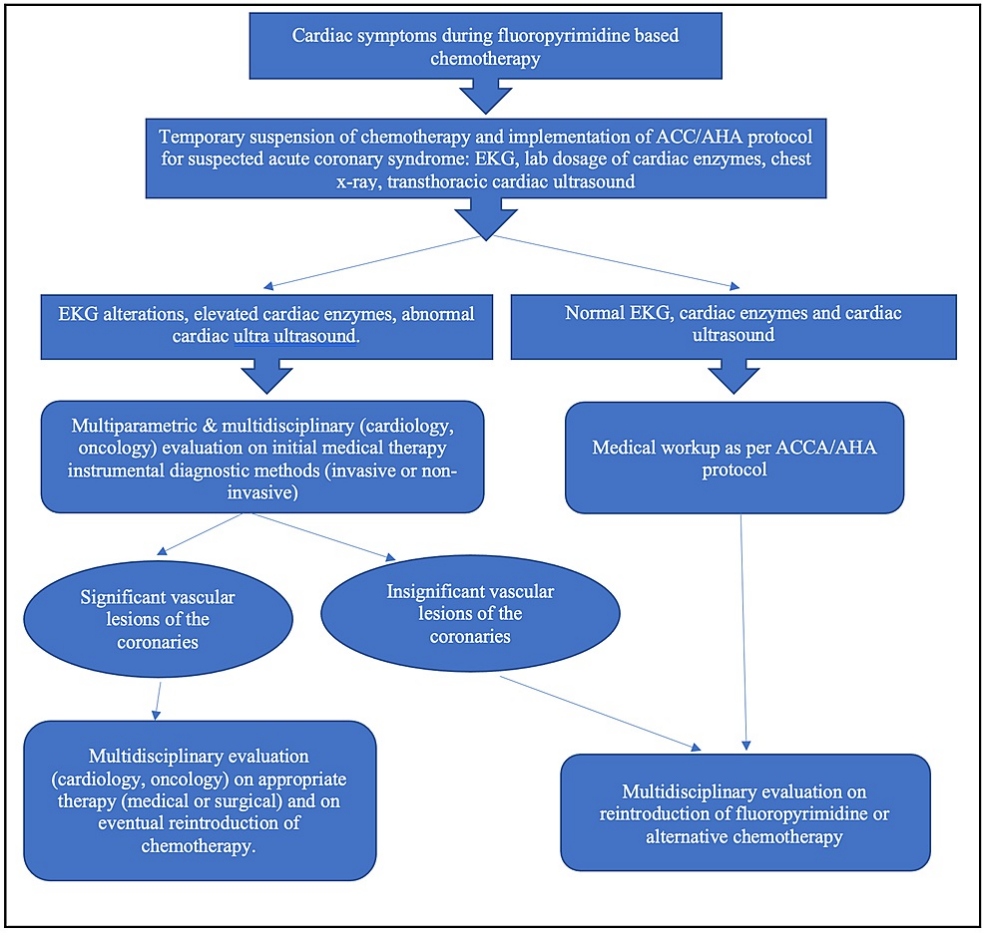
A synthesis of key points on the management of patients with cardiac symptoms in the setting of fluoropyrimidine-based chemotherapy. ACC: American College of Cardiology; AHA: American Heart Association; EKG: electrocardiogram Adapted from: Layoun et al. [17].

All cases of angina in the setting of fluoropyrimidine therapy require the collection of a complete medical history with an evaluation of cardiovascular risk factors and thorough cardiopulmonary physical evaluation. Details noted of the therapy concern the types of drugs used, the means of administration, and the treatment schedule. Each of these could contribute to generating the cardiac adverse effects observed [17]. Immediate EKG helps identify ongoing ischemia and alterations of cardiac rhythms. Cardiac ultrasonography permits the detection of ventricular wall motion aberrations, a sign recorded in 56% of patients with cardiac adverse effects [23]. Patients with angina and EKG alterations receive the attention of the emergency department or a cardiology unit for appropriate further management, as per guidelines of the American College of Cardiology/American Heart Association [17]. The suspension of the chemotherapeutic medication proves beneficial. It often results in a gradual resolution of the symptoms starting a few hours after suspending the treatment, up to a complete solution that may occur days later [23,24,25]. A multidisciplinary strategy helps evaluate these patients to determine how treatment can proceed: reintroducing the chemotherapeutic at lower doses under constant clinical monitoring or applying alternative therapy with biologics. Figure 2 presents a synthesis of the proposed management of these patients.

The toxic events of fluoropyrimidine at the heart level usually occur at the beginning of treatment, as early as within the first 12 hours after drug administration. These effects are unpredictable and may happen at any moment of treatment and sometimes as late as 24 or 48 hours from the onset of therapy. Stopping the drug causes the symptoms to regress rapidly. Considering the highly lethal cardiotoxicity linked to 5-FU treatment, immediately suspending the therapy must be the first thing to do once symptoms present themselves. Following is the empirical management of these symptoms with standard drugs for acute coronary syndrome, as needed, like calcium channel blockers, beta-blockers, and nitrates. The next step is fundamental, for it requires clinical judgment. It consists of determining if the symptoms can be related to 5-FU. This is important because, on the one hand, further exposure to the drug could negatively affect morbidity and mortality. On the other, depriving the patient of life-saving treatment may jeopardize the patient’s possibility of cancer treatment. In making this clinical judgment, the direct relationship between the time elapsed from the administration of 5-FU to the appearance of the cardiac symptoms represents a good hint [6].

If the clinician correlates the cardiotoxicity to 5-FU, a change of chemotherapeutic regimen is the most prudent option. The clinician assumes 5-FU related cardiotoxicity in patients with normal coronary vessels and extremely low-risk factors for coronary artery disease. If available, non-fluoropyrimidine regimens should be considered. The re-use of fluoropyrimidine after cardiotoxicity must entail a detailed discussion between the clinician and the patient on the potential risks and benefits of such a measure. In this case, a prophylactic measure with calcium channel blockers and nitrates associated with meticulous clinical observation could reduce the cardiotoxicity risks [6].

Fluoropyrimidine metabolism and the role of pharmacogenetic testing

5-FU is among the most frequently used chemotherapeutics to treat CRC since its first application in 1957 [26]. It plays a vital role in the production of thymidine to which it is structurally similar; it blocks the enzyme thymidylate synthase and obstructs DNA production. Since this occurs during the S-phase of the cell cycle, 5-FU effectively helps to treat rapidly dividing neoplasia, which develops from epithelium and gland cells [27]. When used with levamisole after surgery, it has contributed to a significant decrease of risks of relapse of stage III CRC by 41% in three years after surgery [28,29]. Notwithstanding their immense beneficial effects, the use of fluoropyrimidines can cause critical adverse reactions in various organs, which may put the life of the patient in danger. These may constitute an obstacle to their use, forcing changes on doses employed or even the suspension of the chemotherapy [19]. As such, it is of great importance to investigate and document possible biological molecules identified in the DPD activity, which can suggest a susceptibility of an individual to fluoropyrimidine-linked adverse reactions [30,31].

Many enzymes participate in the biological breakdown of fluoropyrimidines and form various by-products. The critical step in this process depends on the enzyme DPD. It catalyzes the metabolism of more than 80% of the amount of fluoropyrimidine administered, forming 5-fluoro-5,6-dihydrouracil (5-FDHU). An impediment to the function of DPD results in reduced metabolism of 5-FU, which will result in an augmented risk of toxicity [29,32-34].

85-90% of 5-FU is quickly metabolized in the liver by DPD. Initially, 5,6 dihydrouracil results from this action, and then eventually, alpha-fluoro-beta-ureidopropionic acid and alpha-fluoro-beta-alanine (FBAL). About 90% plasmatic 5-FU is eliminated in the urine in less than 24 hours as FBAL. FBAL is ulteriorly metabolized to fluoroacetate and F-acetate [6,34,35]. F-citrate blocks the Kreb's cycle through its inhibitory action on aconitase, resulting in an accumulation of the metabolite fluoroacetate. This inhibition reduces the cell's capacity to make adenosine triphosphate (ATP), with consequent damage to cardiac tissue and nervous tissue [17]. The genes codifying for DPD may present aberrations. This alteration can compromise the capacity of the enzyme to metabolize 5-FU, rendering the affected individual more prone to adverse cardiac events in different degrees [35]. Understanding such genetic changes helps identify subjects potentially at risk before exposing them to 5-FU.

The main reason a patient might present DPD deficiency seems to result from pathological modifications of the encoding gene *DPYD* and a consequent alteration of the various steps involved in translating the gene, which will affect the protein activity [33,34]. Over 30 variants of the *DPYD* have are known, some of which seem to be accompanied by reduced DPD activity, while a significant number are unclear roles [33,36]. Changes in the structure of the enzyme DPD caused by mutations of the gene *DPYD*, which codifies for the enzyme, results in a complete or partial loss of functionality of the enzyme, influencing the adverse events observed in using fluoropyrimidines [29]. The DPYD*2A (IVS14+1G>A) is a rare, mutated variant of the *DPYD* gene. It is associated with significant and even mortal adverse drug reactions [29], especially in the homozygous state, which presents a total inactivation of the enzyme DPD. When heterozygote, the inactivation is partial and decreased by 50% [37]. Other *DPYD* mutated variants observed to have high chances of toxic adverse reactions linked to fluoropyrimidines are c.1679T>G and c.2846A>T [38]. Some centers implement a protocol based on the screening for these variants associated with the *DPYD* before starting fluoropyrimidine therapy and eventually adjusting the doses of the medication if these variants are present [7].

Besides genotyping, another technique that can permit the identification of DPD deficiency is evaluating its action in the peripheral mononuclear cell (PBMC), which is the phenotypical expression of the enzyme [38]. Because many variants of the *DPYD* gene are not associated with altering the enzyme activity, using one method alone in assessing patients might prove insufficient; applying both genotyping and phenotype variations has been described in studies [39]. The latter method capitalizes on measuring the ratio of dihydrouracil and uracil (UH2/U), done before initiating the chemotherapy and after each cycle of chemotherapy. Table 2 makes a summary of the key points described above.

**Table 2:** Main markers measured to evaluate DPD activity and DPYD variants commonly linked to fluoropyrimidine toxicity. 5-FU: 5-fluorouracil; DPD: dihydropyrimidine dehydrogenase; DHFU: 5-fluoro-dihydrouracil; U: uracil; UH2: dihydrouracil; DPYD: dihydropyrimidine dehydrogenase gene Table created with data from Conti et al. [10].

Substrate	Enzyme	Product	Phenotyping test of DPD activity	DPYD variants associated with toxicity
5-FU	DPD	5-DHFU	Plasmatic 5-FU clearance	c.1236G>A/ HapB3 c.1679T>G c.1905+1G>A c.2846A>T
Uracil (U)	DPD	Dihydrouracil (UH2)	Uracil test dose UH2/U in plasma	

Limitations

Since there was no restriction of publication of the included articles, we consulted a few older studies to synthesize the current review. We exclusively selected only studies that we could find published in the English language; hence, we cannot exclude the possibility of missing out on relevant articles published in other languages.

## Conclusions

CRC is a significant cause of death by cancer worldwide, the second most frequent. Surgery is fundamental but often insufficient to treat advanced stages of CRC. Adjuvant and neoadjuvant chemotherapy is necessary to treat higher stages of the disease. In this regard, 5-FU and its prodrug and oral equivalent, capecitabine, have significantly reduced mortality by improving prognosis in CRC patients. A small percentage of individuals present mutations of the gene that codifies pyrimidine dehydrogenase. These persons risk significant toxicity when exposed to pyrimidine-based chemotherapy. This possibility influences the continuation of the regime of therapy. It inevitably means prolonged hospital admission with necessary diagnostic workouts and treatment when cardiotoxicity occurs and increases healthcare costs. Some cancer centers consider pre-therapy testing to identify mutated variants of the *DPYD* before initiating therapy. This approach is not yet a common practice for CRC, which has a treatment (5-FU) that can have potentially lethal cardiac adverse effects. Considering the significant advances made in CRC management, like primary and secondary preventive measures, we believe further improvements require addressing the adverse events related to 5-FU linked chemotherapy more. Identifying patients who bear *DPYD* variants linked with increased cardiotoxicity and better management of their therapeutic regimes in a multidisciplinary fashion reduces these risks of adverse pyrimidine-related adverse reactions. These interventions lower the chances of hospitalizations and its costs and initiate better patient care for CRC, which is a significant cause of cancer-related deaths.
